# Genetic diversity and occurrence of the F129L substitutions among isolates of *Alternaria solani* in south-eastern Sweden

**DOI:** 10.1186/s41065-016-0014-0

**Published:** 2016-09-23

**Authors:** Firuz Odilbekov, Eva Edin, Larisa Garkava-Gustavsson, Helena Persson Hovmalm, Erland Liljeroth

**Affiliations:** 1 Department of Plant Protection Biology, Swedish University of Agricultural Sciences, P.O. Box 102, SE-230 53 Alnarp, Sweden; 2 Department of Forest Mycology and Plant Pathology, Swedish University of Agricultural Sciences, P.O. Box 7026, SE-750 07 Uppsala, Sweden; 3 Department of Plant Breeding, Swedish University of Agricultural Sciences, P.O. Box, 101, SE-230 53 Alnarp, Sweden

**Keywords:** AFLP, Early blight, Potato, Strobilurin, Sensitivity

## Abstract

**Background:**

Early blight, caused by the fungus *Alternaria solani,* occurs on potato mainly in the south-eastern part of Sweden, but also in other parts of the country. The aim of this study was to investigate the genetic diversity of *A. solani* populations from different potato growing regions in south-eastern Sweden using AFLP marker analysis. In addition, the cultured isolates were examined for substitutions in the gene encoding cytochrome *b*, associated with loss of sensitivity against QoI fungicides.

**Results:**

Nei's gene diversity index for the Swedish populations of *A. solani* revealed a gene diversity of up to 0.20. Also genetic differentiation was observed among populations of *A. solani* from different locations in south-eastern Sweden. The mitochondrial genotype of the isolates of *A. solani* was determined and both known genotypes, GI (genotype 1) and GII (genotype 2), were found among the isolates. The occurrence of the F129L substitution associated with a loss of sensitivity to strobilurins was confirmed among the GII isolates. In vitro conidial germination tests verified that isolates containing the F129L substitution had reduced sensitivity to azoxystrobin and, at a lower extent, to pyraclostrobin.

**Conclusions:**

Genetic diversity was relatively high among isolates of *A. solani* in south-eastern part of Sweden. F129L substitutions, leading to reduced sensitivity to strobilurins, have been established in field populations, which may have implications for the future efficacy of QoI fungicides.

## Background

Several fungal species within the genus *Alternaria* are known as destructive plant pathogens [1] causing severe damage leading to economic losses for growers. *Alternaria solani* is an asexual plant pathogenic species that causes early blight on potato (*Solanum tuberosum* L.), and other members of the *Solanaceae* family. The disease may result in large crop losses in many potato and tomato producing areas worldwide [2]. The pathogen mostly infects the foliage and produces dark brown lesions with concentric rings that enlarge, coalesce and eventually cause leaf death [3]. The fungus may also infect tubers during storage in some areas, but relatively little research has been carried out on tuber diseases caused by *Alternaria* sp. [4, 5].

Early blight is a common fungal disease in Swedish potato fields and during the last decade a number of reports have stressed that the disease is an increasing problem in the south-eastern part of the country, especially in starch potato crops. Both *A. solani* and *A. alternata* have been detected in the field, but *A. solani* was found more often [6]. Further investigations confirmed that early blight in south-eastern Sweden is mainly caused by *A. solani* [7].

Effectiveness of host resistance and fungicide application can, to a great extent, be influenced by the genetic variation of pathogens [8]. Therefore, to improve plant disease management, knowledge about the genetic structure of the pathogen population should be taken into consideration [9]. Several studies point towards high genetic variation among isolates of *A. solani*, even though it is considered as an asexually reproducing fungus. Isozyme analyses revealed high genetic variation among isolates of *A. solani* from both potato and tomato in the USA [10] and this was subsequently confirmed using RAPD marker analysis [11]. Similarly, high genetic variability among South African isolates obtained from potato was observed by population analysis using random amplified microsatellite markers (RAMS) [12] and among Chinese isolates from potato using Amplified fragment length polymorphism (AFLP) fingerprints [13]. However, in Sweden and other Nordic countries no studies of *A. solani* populations have been reported.

AFLP remains a powerful and highly reproducible PCR-based technique for DNA-fingerprinting. Since this method does not require prior knowledge of genomic sequence and produces large number of polymorphic loci, it is still one of the most commonly used PCR based methods for genetic diversity analysis. This method has been used in several studies of genetic diversity in *Alternaria* species [9, 13, 14].

The most common way of controlling early blight in Swedish potato production today is to treat the crop with Qol fungicides (strobilurins). This method has so far been efficient in controlling early blight [6]. However, strobilurins have been reported to show reduced efficacy against species of *Alternaria* in some parts of the USA [15–18]. Strains of *A. solani* that display reduced sensitivity to strobilurins have nucleotide substitutions in the amino acid codon at position 129 (referred to as F129L, phenylalanine has changed to leucine) in the gene encoding cytochrome *b* [17]. Recently, it has been discovered that *A. solani* in Europe carries two types of mitochondrial DNA. Populations carrying these two DNA types are referred to as genotype 1 (GI) and genotype 2 (GII) [19]. The latter resembles the American population of *A. solani* and can only be distinguished by PCR with special primers [15]. Analysis of *Alternaria* populations from Germany revealed the presence of the F129L substitution and the frequency of this substitution increased over the years [19]. Isolates that carried the F129L substitution had reduced in vitro sensitivity to Qol fungicides. In Sweden, observations of reduced field efficacy of strobilurins have been reported during the last few years, especially in the area around Kristianstad (personal communication with growers, advisors and the Swedish Board of Agriculture).

The objectives of the present study were to: 1) examine the genetic diversity within and among populations of *A. solani* from two potato growing regions in south-eastern Sweden by applying AFLP marker analysis; 2) examine the cultured isolates for substitutions in the gene encoding cytochrome *b* that are associated with a loss of sensitivity to stroilurins.

## Methods

### Collection, isolation and identification of fungal cultures

**Fig. 1:**
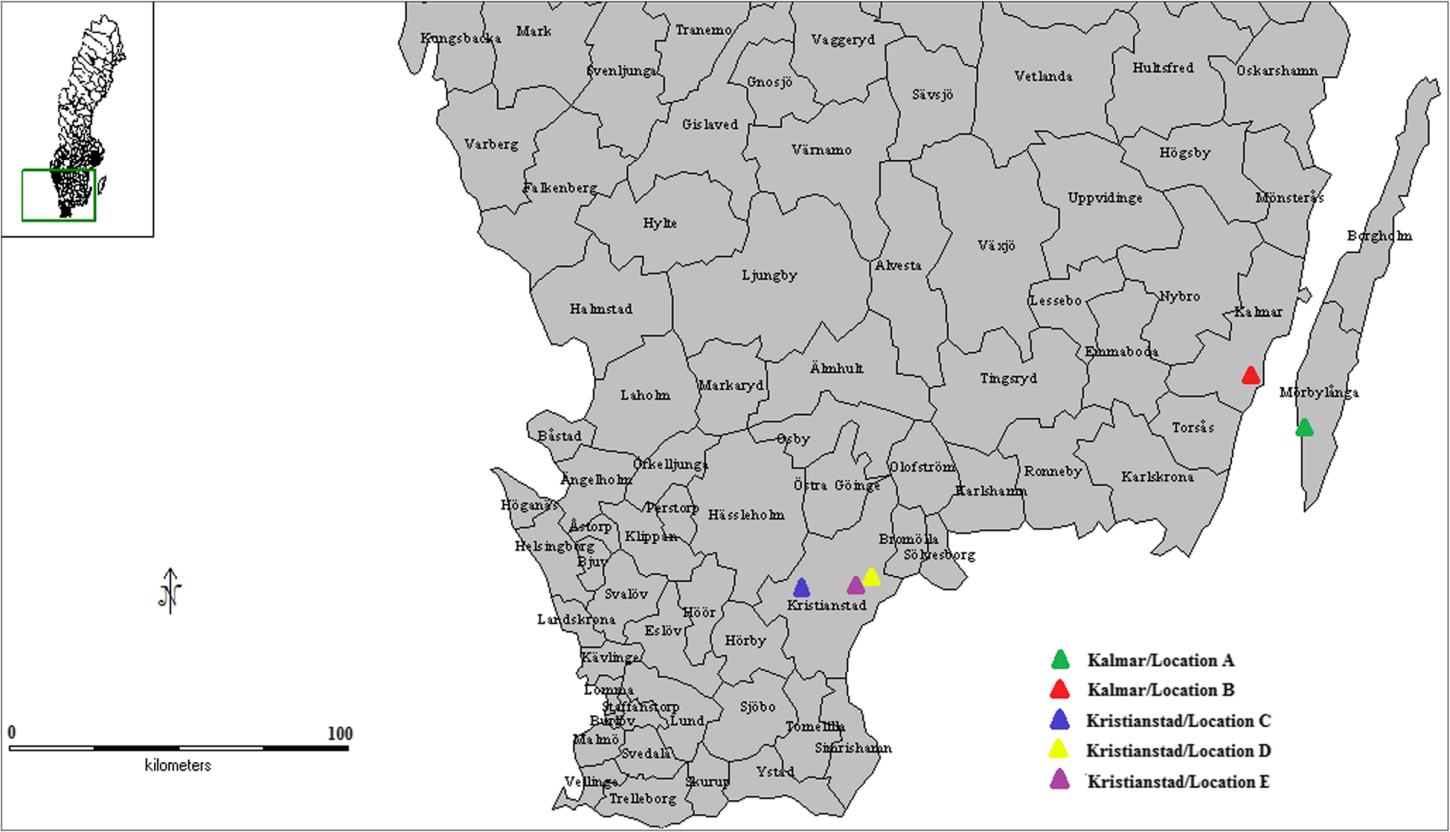
Map of southern Sweden showing the location where the isolates of *Alternaria solani* were sampled

Leaflets with symptoms resembling early blight were collected in starch potato fields in two regions (Kalmar/Öland and Kristianstad) of South-eastern Sweden during September 2011. Two fields were sampled in the Kalmar/Öland region and three in the vicinity of Kristianstad (Fig. 1). The sampling was performed in four rows with eight rows in between. In each row, samples were collected at six points, approximately 10 m apart. The leaflets were placed in small paper bags and air dried. The sampled fields had been treated at least once with strobilurins, either in the second or the fourth week of July, prior to sampling. The majority of the fields were treated twice.

Sections 3–4 mm around the edge of lesions were cut and surface sterilized in 1 % sodium hypochlorite for 3 min followed by two times washing in sterile distilled water. The leaf discs were placed on water agar containing a broad-spectrum antibiotic (chlorotetracycline, 100 μg mL^−1^) and stored in darkness at room temperature for 3–4 days. Single conidium was picked directly from the infected leaf tissue with a tiny needle under a stereo-microscope and placed on new potato dextrose agar for germination. Species identification was performed morphologically and identifications of *A. solani* were confirmed with PCR based methods [18, 20]. In addition all isolates were also checked with specific primers developed for the closely related species *A. tomatophila* [21]. All reactions were performed in duplicates with a positive and a negative control. The primers for identification of *A. solani* GI [20] amplify both genotypes but the PCR-product of GII is shorter and the normal amino acid codon sequence around position 129 is absent. Fifty-five isolates of *A. solani* representing different regions, fields and rows within fields (three isolates per row from four rows per field) were selected for analysis (Table 1). Four isolates of *A. alternata* from Sweden were included in the UPGMA analysis as comparison (see below).

**Table 1:** Geographic origin and mitochondrial genotype of the *Alternaria solani* isolates used in this study

No	Species	Geographic region	Location	GI	GII	GII^a^	Abbreviation
1	*A. solani*	Kalmar	A	-	+	-	ASKLA1
2	*A. solani*	Kalmar	A	+	-	-	ASKLA2
3	*A. solani*	Kalmar	A	+	-	-	ASKLA3
4	*A. solani*	Kalmar	A	+	-	-	ASKLA4
5	*A. solani*	Kalmar	A	n.d	n.d	n.d	ASKLA5
6	*A. solani*	Kalmar	A	-	+	-	ASKLA6
7	*A. solani*	Kalmar	A	-	+	-	ASKLA7
8	*A. solani*	Kalmar	A	+	-	-	ASKLA8
9	*A. solani*	Kalmar	A	+	-	-	ASKLA9
10	*A. solani*	Kalmar	A	-	+	-	ASKLA10
11	*A. solani*	Kalmar	A	+	-	-	ASKLA11
12	*A. solani*	Kalmar	A	+		-	ASKLA12
13	*A. solani*	Kalmar	B	-	+	-	ASKLB1
14	*A. solani*	Kalmar	B	+	-	-	ASKLB2
15	*A. solani*	Kalmar	B	+	-	-	ASKLB3
16	*A. solani*	Kalmar	B	+	-	-	ASKLB4
17	*A. solani*	Kalmar	B	+	-	-	ASKLB5
18	*A. solani*	Kalmar	B	+	-	-	ASKLB6
19	*A. solani*	Kalmar	B	+	-	-	ASKLB7
20	*A. solani*	Kalmar	B	+	-	-	ASKLB8
21	*A. solani*	Kalmar	B	+	-	-	ASKLB9
22	*A. solani*	Kalmar	B	+	-	-	ASKLB10
23	*A. solani*	Kalmar	B	+	-	-	ASKLB11
24	*A. solani*	Kalmar	B	+	-	-	ASKLB12
25	*A. solani*	Kristianstad	C	n.d	n.d	n.d	ASKRLC1
26	*A. solani*	Kristianstad	C	+	-	-	ASKRLC2
27	*A. solani*	Kristianstad	C	+	-	-	ASKRLC3
28	*A. solani*	Kristianstad	C	+	-	-	ASKRLC4
29	*A. solani*	Kristianstad	C	+	-	-	ASKRLC5
30	*A. solani*	Kristianstad	C	-	+	-	ASKRLC6
31	*A. solani*	Kristianstad	C	n.d	n.d	n.d	ASKRLC7
32	*A. solani*	Kristianstad	C	+	-	-	ASKRLC8
33	*A. solani*	Kristianstad	C	+	-	-	ASKRLC9^b^
34	*A. solani*	Kristianstad	C	+	-	-	ASKRLC10
35	*A. solani*	Kristianstad	C	+	-	-	ASKRLC11
36	*A. solani*	Kristianstad	C	+	-	-	ASKRLC12
37	*A. solani*	Kristianstad	D	-	-	+	ASKRLD1
38	*A. solani*	Kristianstad	D	-	-	+	ASKRLD2
39	*A. solani*	Kristianstad	D	-	-	+	ASKRLD3^b^
40	*A. solani*	Kristianstad	D	-	-	+	ASKRLD4
41	*A. solani*	Kristianstad	D	n.d	n.d	n.d	ASKRLD5
42	*A. solani*	Kristianstad	D	-	-	+	ASKRLD6^b^
43	*A. solani*	Kristianstad	D	n.d	n.d	n.d	ASKRLD7
44	*A. solani*	Kristianstad	D	-	-	+	ASKRLD8
45	*A. solani*	Kristianstad	D	-	-	+	ASKRLD9
46	*A. solani*	Kristianstad	D	-	-	+	ASKRLD10
47	*A. solani*	Kristianstad	D	-	-	+	ASKRLD11
48	*A. solani*	Kristianstad	D	-	-	+	ASKRLD12
49	*A. solani*	Kristianstad	E	+	-	-	ASKRLE1
50	*A. solani*	Kristianstad	E	n.d	n.d	n.d	ASKRLE2
51	*A. solani*	Kristianstad	E	+	-	-	ASKRLE3
52	*A. solani*	Kristianstad	E	+	-	-	ASKRLE4
53	*A. solani*	Kristianstad	E	+	-	-	ASKRLE5
54	*A. solani*	Kristianstad	E	n.d	n.d	n.d	ASKRLE6
55	*A. solani*	Kristianstad	E	+	-	-	ASKRLE7

*GI* genotype I, *GII* genotype II, ^a^genotype II with F129L substitution, ^b^not included in diversity study, *n.d* not determined

### DNA extraction

For DNA extraction, each isolate was grown in a liquid medium containing 10 gL^−1^ of sucrose, 2 gL^−1^ of L-asparagin, 2 gL^−1^ of yeast extract, 15 mM KH_2_PO_4_, 0.4 mM MgSO_4_ × 7H_2_O, 1.5 μM ZnSO_4_ × 7H_2_O, 1.8 μM FeCl_3_ × 6 H_2_O, and 2.5 μM MnCl_2_ x H_2_O in Erlenmeyer flasks under continuous agitation (60 rpm) at room temperature [22]. After eight days, the mycelium was washed with sterile distilled water, transferred to filter paper and freeze dried. Genomic DNA Purification Kit (Fermentas Lithuania) was used to extract the total genomic DNA. Depending on the size of the DNA pellet, 30 to 50 μL of ddH_2_O with RNase was added and the pellet was re-suspended at 37 °C for 2 h and subsequently stored at 4 °C. The quality of DNA was determined by electrophoresis using 1 % agarose gel containing ethidium bromide and the final concentration was adjusted to 100 ng/μl using a Nanodrop® ND-1000 spectrophotometer (NanoDrop Technologies, Inc. DE, USA).

### AFLP analysis

The AFLP analysis was performed using an AFLP Microbial Fingerprinting Kit (Applied Biosystems, CA, USA) based on a modified manufacturer’s protocol [23]. Genomic DNA of each isolate was digested with two restriction enzymes (*Eco*RI and *M*seI), ligated to oligonucleotide adapters and pre-amplification was performed. The selective amplification was performed using seven labelled primer combinations: E + AC⁄M + A, E+ AC⁄M + G, E + AC⁄M + C, E + AA⁄M + A, E + AA⁄M + G, E + AA⁄M+ C and E + AT⁄M + A [9]. The main amplified PCR products were multiplexed into panels using different fluorescent labels and analysed on an ABI 3730 capillary DNA analyser (Applied Biosystems) at the University of Copenhagen, Denmark. The results were visualized and analysed using Genemarker (Softgenetics®, PA, USA). Each individual band was scored manually using both the gel image and the peak height. In both cases, default settings in Genemarker were applied for detection of bands with the recommended threshold intensity of 100. Bands between 60 and 500 base pairs were scored as either present “1” or absent “0”. Only bands that could be scored unambiguously were included in the AFLP analysis.

### Data analysis

Genetic diversity was calculated by the number and percentage of polymorphic loci, Shannon’s information index (I) and Nei’s gene diversity (H). For each population, Nei’s gene diversity (H) and Shannon’s index (I) were calculated for each locus and then averaged over all loci. Calculations of these parameters were performed using POPGENE version 1.32. A dendrogram was obtained by cluster analysis of all isolates using the unweighted pair group method with arithmetic means (UPGMA) [24], similarity coefficient (SAHN procedure in the NTSYS pc 2.2 statistical package). The FreeTree software [25] was applied for statistical support of dendrogram branches with 1000 bootstrapping samples. Principal coordinate analysis (PCoA) was performed to obtain a graphic representation of the relationship among the 52 isolates of *A. solani,* since some of the isolates did not give any results. Calculations were made using the procedures in the NTSYS pc 2.2 statistical package. Analysis of molecular variance (AMOVA) was carried out by using Arlequin 3.0 [26]. The number of permutations for significance tests was set at 1000 for all analyses.

### Cytochrome *b* substitutions

To detect any substitution in the gene encoding cytochrome *b* associated with loss of sensitivity to strobilurins, the region was amplified using PCR and then sequenced. DNA from all samples determined as *A. solani* was amplified using specific primers. For the GI genotype the procedure of Edin [20] was followed and for the GII genotype primers developed by Pasche et al. [15] were used for the PCR amplifications. The PCR solution of 50 μl contained 0.25 ng DNA μL^−1^, 0.75 mM MgCl_2_ (final concentration), 0.2 mM dNTP, 0.2 μM of each primer, 0.03 U μL^−1^ ThermoRed DNA Polymeras (Saveen & Werner AB) and corresponding reaction buffer. The PCR conditions were 96 °C for 5 min, 40 cycles of 30 s at 96 °C, 30 s at 60 °C (GI) or 54 °C (GII) and 30 s at 72 °C, followed by a 5 min extension. The success of the PCR amplifications was analysed using electrophoresis (1 % agarose gel stained with Nancy-250 (Sigma-Aldrich, MO USA). The products were purified using Agencourt AMPure XP (Beckman Coulter, MA, USA) according to the manufacturer’s protocol and sequenced at Macrogen Inc. Seoul, South Korea. The procedures were repeated for those isolates with conflicting results.

### In vitro sensitivity assay

Azoxystrobin and pyraclostrobin (analytical standard, Sigma) were dissolved in 1 mL acetone to a concentration of 100 mg mL^−1^ and used as stock solution. Petri dishes with water agar containing different concentrations of azoxystrobin or pyraclostrobin (0, 0,01 0,1 1 and 10 μg mL^−1^) were prepared. The agar also contained 100 mg L^−1^ salicylhydroxamic acid (SHAM). Petri dishes with SHAM but no azoxystrobin/pyraclostrobin were used as a control. The final concentration of acetone in all media was 0.1 % (*v/v*). Spores of *A. solani* were produced [27] and the spore suspension was adjusted to 2 × 10^4^ conidia mL^−1^. Fifty microliters of conidial suspension of each isolate was spread across the agar plates, containing different concentrations of azoxystrobin/pyraclostrobin (two replicate plates) and on control plates (two replicate plates). Plates were incubated in a growth chamber at a temperature of 28 °C under continuous light for 5 h and germination of 100 conidia was evaluated microscopically at 100 x magnification. For each isolate, the concentration that effectively inhibited germination of 50 % of the conidia relative to the untreated control (EC_50_) was calculated. Then the plates were incubated again for another 10 h (in total, 15 h) and the germination rate was measured again. Five wildtype isolates and five isolates with F129L substitution from 2011 were evaluated and the test was repeated once. In addition, ten isolates with F129L substitution obtained from Kristianstad location E in 2014 were evaluated as comparison.

## Results

### Genetic diversity

In total, 271 AFLP bands were observed using the seven selected primer combinations and close to 100 % of the bands produced were polymorphic (Table 2). The degree of polymorphism for *A. solani* isolates ranged from 36.2 to 98.4 %. The primer combinations E + AA/M + A, E + AA/M + C and E + AT/M + A gave higher percentages of polymorphism in *A. solani* compared to the other primer combinations (Table 2).

**Table 2:** Number of amplified AFLP fragments and degree of polymorphism among isolates of *Alternaria solani* with different EcoRI/MseI primers

Primer combination	(*n* = 52)	
	Total^a^	%pol^b^
*E + AA/M + A*	68	94,4
*E + AA/M + C*	61	98,4
*E + AA/M + G*	46	83,6
*E + AC/M + A*	31	70,4
*E + AC/M + C*	17	36,2
*E + AC/M + G*	16	42,1
*E + AT/M + A*	32	91,4
Total	271	73,8

^a^Total number of fragments observed

^b^Percentage of total fragments that were polymorphic

A comparison of the Nei's gene diversity index for the five Swedish populations of *A. solani* revealed that the gene diversity was lowest in the Kristianstad/location D population (0.080) and highest in the Kalmar/location A population (0.182) (Table 3). Also for the Shannon’s index, Kristianstad/location D had the lowest value (0.120) whereas Kalmar/location A had the highest (0.273). Comparisons between the two Swedish regions revealed that the Kalmar region had a higher level of diversity than the Kristianstad area.

**Table 3:** Gene diversity estimators for populations of *Alternaria solani* from different regions in Sweden based on results of seven amplified fragment length polymorphism (AFLP) primer pair combinations

All location	NI	NPL	PPL	H	I
Kalmar/Location A	12	180	51,0	0,182	0,273
Kalmar/Location B	12	175	49,6	0,155	0,238
Kristianstad/Location C	11	195	55,2	0,167	0,260
Kristianstad/Location D	10	81	23,0	0,080	0,120
Kristianstad/Location E	7	140	39,7	0,151	0,223
All Kalmar locations	24	244	69,1	0,206	0,317
All Kristianstad locations	28	219	62,0	0,166	0,259

*NI* number of isolates, *NPL* number of polymorphic loci, *PPL* percentage of polymorphic loci, *H* Nei’s gene diversity, *I* Shannon’s information index

**Fig. 2:**
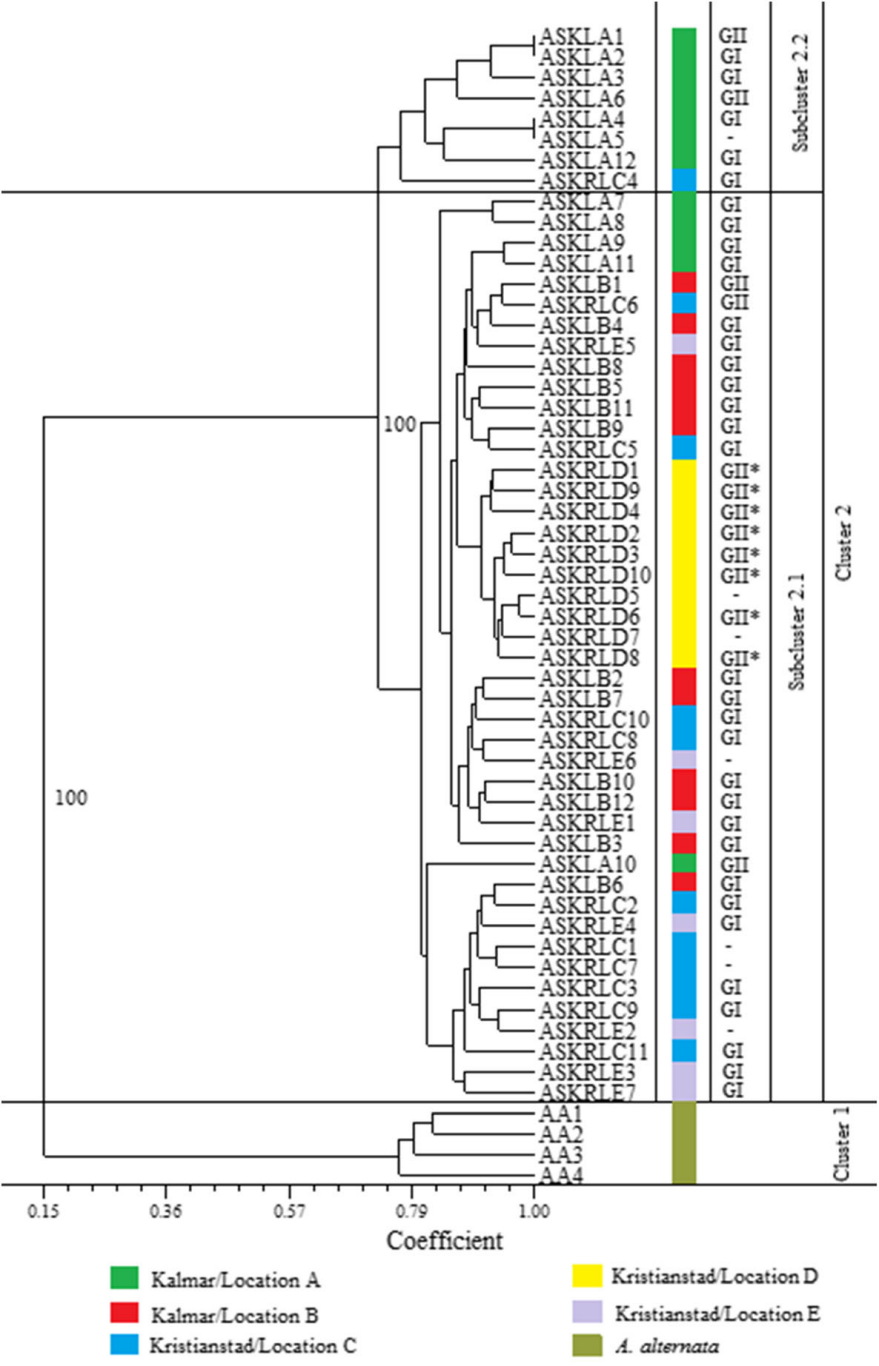
Dendrogram from cluster analysis (UPGMA) based on Dice genetic similarity for *Alternaria solani* isolates from Sweden. The values at the branches are the bootstrap values generated by 1000 re-samplings. The colour shows different locations where the samples were collected. GI = genotype I, GII = genotype II, GII* = genotype II with F129L substitution. A few *A. alternata* isolates were included as an outgroup

UPGMA cluster analysis (see dendrogram; Fig. 2) clearly separated the two species into two main clusters. This separation was supported by a high bootstrap value (100 %). There were two distinct sub-clusters of *A. solani* isolates within cluster 2 in 100 % of the 1000 bootstrapped trees. The first sub-cluster (2.1) consisted of 44 isolates from all five locations. Isolates from Kristianstad/location D grouped together but with poor statistical support. Sub-cluster 2.2 comprised eight *A. solani* isolates, including seven isolates from Kalmar/location A and one isolate from Kristianstad/location C. The Kalmar/location A was the only field where the isolates were highly separated genetically.

**Fig. 3:**
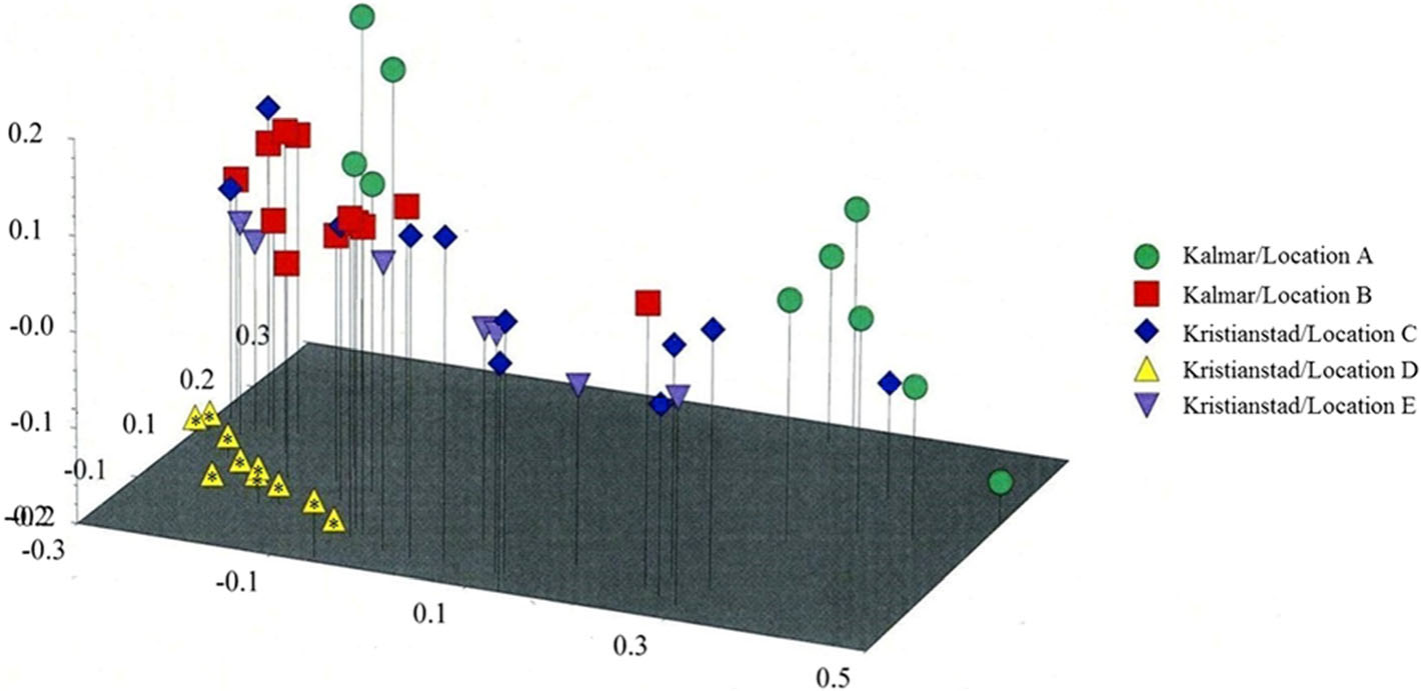
Principal coordinate analysis (PCoA) of 52 isolates of *Alternaria solani* from five locations in Sweden based on AFLP data. * = GII with F129L mutation

Principal coordinates analysis was performed to further evaluate relatedness among the *A. solani* isolates. The first three principal components explained 28, 9.6 and 6 % of the total variation, respectively. Thus, the three-dimensional plot (Fig. 3) summarizes 43.6 % of the total variation in all *A. solani* isolates. Isolates from Kristianstad/location D grouped more closely together in this analysis compared to the UPGMA, which indicates high genetic similarity of the isolates from this location. All isolates, except one, from Kalmar/location B also grouped closely together. AMOVA was used to partition the total genetic variance within and among population components (Table 4). The percentage of variation among populations was 20 % (Fst = 0,20, *P* < 0.0001) and a much higher proportion of variation was observed within populations (80 %).

**Table 4:** Analysis of molecular variances among the investigated populations of *Alternaria solani* in Sweden

Source of variation	d.f	Sum of squares	Variance components	Variation (%)	Fst	Probability
Among Populations	4	416	7.27 Va	20	0.20	Va and Fst = 0.0000
Within populations	47	1366	29.0 Vb	80		

### Cytochrome *b* mutants and sensitivity test

Both genotypes, GI and GII, were found in three out of five fields (Table 1). None of the GI genotypes carried the F129L substitution, while the majority of the GII isolates did. Four wild type GII isolates were found at location A in Kalmar, while a single wild type GII isolate was found at location B in Kalmar and location C in Kristianstad, respectively. At location D in Kristianstad, all ten investigated isolates were GII with the F129L substitution. This was the only field where F129L substitution was found 2011. However, in 2014 isolates carrying F129L were also found in Kristianstad location E.

**Fig. 4:**
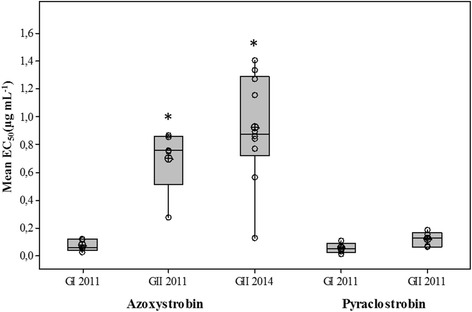
Mean of EC_50_ values for wild type (GI) (*n* = 5, 2011) and F129L substitution isolates (GII) (*n* = 5, 2011 and *n* = 10, 2014) of *Alternaria solani* obtained from in vitro sensitivity tests of azoxystrobin and pyraclostrobin after 5 h incubation. * = significantly different from wild type 2011 (*p* < 0.05) according to two sample t-tests using Minitab 16.2.4 statistical software

All *A. solani* isolates with the F129L substitution tested in vitro were less sensitive to azoxystrobin (Fig. 4). Means of EC_50_ values, based on germination rates 5 h after inoculation for isolates from 2011 and 2014 that contained the substitution were 0.7–0.9 μg mL^−1^ respectively, while the mean of the wild type isolates was 0.07 μg mL^−1^. Overall, comparing mean EC_50_ values, wild type isolates were 10-fold more sensitive compared to isolates containing F129L substitution from 2011 and 13-fold more sensitive in comparison to isolates from 2014 (Fig. 4). The shift to reduced sensitivity was less pronounced with pyraclostrobin compared to azoxystrobin and wild type isolates from 2011 were 2-fold more sensitive to pyraclostrobin than isolates with the F129L substitution (Fig. 4). However, a much higher degree of differentiation in sensitivity between wildtype and mutants was found 15 h after inoculation at concentrations 0.1, 1 and 10 μg mL^−1^ (Table 5).

**Table 5:** In vitro sensitivity test of *Alternaria solani* wild type and F129L substitution isolates collected in 2011. Percentage of spores germinated after 15 h of incubation on media containing azoxystrobin or pyraclostrobin

Isolate ID	Genotype	*Azoxystrobin, μg/ml*					*Pyraclostrobin, μg/ml*				
		*0*	*0,01*	*0,1*	*1*	*10*	*0*	*0,01*	*0,1*	*1*	*10*
ASKLA2	GI	100	95	76	61	5	100	98	69	19	0
ASKLB3	GI	100	95	93	54	4	100	100	48	7	0
ASKLB4	GI	100	96	73	9	0	100	97	21	1	0
ASKLB8	GI	100	98	93	41	2	100	100	53	16	0
ASKLB9	GI	100	95	87	65	0	100	100	73	23	0
ASKRLD1	GII^a^	100	100	99	92	78	100	100	79	30	0
ASKRLD2	GII^a^	100	100	100	98	83	100	100	89	67	0
ASKRLD4	GII^a^	100	100	100	94	78	100	100	91	63	2
ASKRLD9	GII^a^	100	100	99	99	80	100	100	94	68	9
ASKRLD10	GII^a^	100	100	99	93	65	100	100	69	41	0

^a^Isolate contains F129L substitution

### Correlation between genetic structure, mitochondrial genotype and presence of cytochrome *b* mutants

In general, there was no obvious correlation between the geographical origin of the *A. solani* isolates and the genetic structure. However, in one location, Kristianstad D, all the isolates were GII with the F129L substitution and they grouped closely together in the PCoA plot (Fig. 3). The isolates from the other populations were scattered in the PCoA and showed no clear pattern of correlation between mitochondrial genotype and genetic structure. There was no correlation between mitochondrial genotype and clustering (Fig. 2).

## Discussion

The level of genetic diversity among isolates of *A. solani* from South-eastern Sweden was found to be relatively high for a species assumed to only have asexual reproduction. Previous studies of these species have shown genetic distinctness between populations of *A. solani* and *A. alternata* infecting potato [10] and both tomato and potato in the USA [11], Cuba [14] and Brazil [9]. The level of diversity in *A. solani* was slightly lower compared to the previously mentioned studies as indicated by the diversity indexes in the present study. A possible explanation could be that other studies used isolates from different Solanaceous hosts while we investigated only samples from potato. Different results may also be due to different sample sizes.

Both mitochondrial genotypes as well as the F129L substitution in the cyt *b* gene occur within *A. solani* populations in Sweden. In Europe, *A. solani* isolates containing the F129L substitution were first found in Germany. Isolates possessing the substitution also displayed a shift in sensitivity to different strobilurins in in vitro spore germination assays [19]. None of the Swedish GI isolates carried the F129L substitution, while the F129L substitution was observed in many of the GII isolates. Results from the present study revealed that isolates carrying the F129L substitution exhibited a reduction in sensitivity to azoxystrobin as well as to pyraclostrobin in in vitro spore germination tests (Fig. 4). The shift to reduced sensitivity also tended to be more pronounced in isolates with the F129L substitution collected in 2014 compared to isolates from 2011, indicating that application of QoI fungicides has resulted in reduced sensitivity of the isolates over time. These results are in accordance with the previous study [19], which also reported that isolates carrying this substitution had reduced in vitro sensitivity to QoI fungicides, especially against azoxystrobin.

EC_50_ values are commonly calculated based on spore germination rates at 5 h after inoculation. However, we found a better differentiation between the wildtype and isolates harboring the F129L substitution after 15 h. At the highest concentrations there was a clear difference in germination rate (Table 5). In addition, a field trial during 2014 clearly showed that significant disease control was not obtained by using azoxystrobin only against early blight (unpublished).

There was a significant genetic differentiation (Fst = 0.200, *P* < 0.0001) among the *A. solani* populations from the different locations. UPGMA sub-cluster 2.2 that deviated from the other *A. solani* clusters (Fig. 2) was dominated by isolates from Kalmar location A, suggesting that part of the population on the island in the Kalmar region is genetically different. Fst values between 0.15 and 0.25 reportedly represent moderately high genetic differentiation [28]. The mitochondrial DNA and genomic DNA did not have any apparent correlation, since the F129L locus was not associated with any specific AFLP-locus and both genotypes, GI and GII, were found in all clusters. In one of the Kristianstad locations (location D) *A. solani* isolates that carried the F129L substitution grouped closely together (Fig. 3). However, in the other clusters, isolates from different locations grouped together indicating that migration may have taken place.

*Alternaria solani* is not known to undergo sexual recombination. However, genetic recombination in different putatively asexual fungal populations has been reported [29–32]. According to [29–32], these fungi may have an alternative mechanism for promoting recombination of genomes. Recombination events within *A. alternata* subpopulations were observed suggesting that a non-meiotic mechanism of recombination, i.e. a parasexual cycle, may be operating [32]. Cytological and morphological studies suggested that heterokaryosis could occur in *A. solani* [33]. Heterokaryosis can be preserved or lost during further cell divisions. Also nuclear migration could occur through septal pores between cells of conidia, conidiophores and mycelia, allowing dissociation of unlike nuclei leading to homokaryosis, or re-establishment of heterokaryosis [33]. Therefore, even isolates from single conidia or hyphal tips could be genetically diverse.

Another possible explanation for the high level of genetic variation among the isolates, including the presence of F129L, is natural mutations that occur spontaneously. The pathogen produces abundant numbers of spores in a relatively short period of time, and mutations may play an important role in generating diversity [10, 12, 34]. Natural mutations may occur more frequently in asexually reproducing isolates than in sexually reproducing ones [35]. Several studies have reported mutations in *A. solani* populations in the USA, especially in mitochondrial DNA [15, 36] and in Germany [19]. The high usage of fungicides most likely explains why the genetically diverse populations have the same mutation. The mutation is being strongly selected for. However, to quantify the mutation rate within *A. solani* the complete genome of the species must be sequenced.

Understanding the genetic diversity of *A. solani* is a base for optimizing disease management strategies against early blight. Further genetic studies on the pathogen involving sampling of a larger number of (sub)-populations covering a wider geographic range would enhance our understanding of population structure, levels of genetic variation and migration patterns in Sweden and elsewhere.

In practical agriculture, fungicide resistance is one of the main problems with chemical pest management, and one that has become an issue in the control of *Alternaria* spp. As a result of the frequent applications of QoI fungicides in potato fields in USA [17] and Germany [19] *A. solani* isolates with reduced sensitivity to strobilurins have increased in frequency. Today, all farmers are expected to use different anti-resistance strategies, e.g. alternating fungicides with different modes of action, to prolong the effectiveness of the fungicides. Application of complex or multisite mode of action fungicides in spray programmes decreases the risk that fungicide resistance develops in the pathogen population (www.frac.info).

## Conclusion

Our results provide the first insight into the level of genetic variation and the presence of F129L substitutions associated with loss of sensitivity against strobilurins among populations of *A. solani* from different locations in south-eastern Sweden. AFLP marker analysis indicates that genetic diversity among the studied isolates is relatively high and that the isolates showed a significant genetic differentiation. In addition genetic analysis of the isolates confirmed the presence of F129L substitutions, which is associated with loss of sensitivity against strobilurins.

## Abbreviations

AFLP: Amplified fragment length polymorphism
AMOVA: Analysis of molecular variance
EC50: Half maximal effective concentration
F129L: An amino acid substitution of phenylalanine with leucine at position 129
GI: Genotype 1
GII: Genotype 2
H: Nei’s gene diversity
I: Shannon’s information index
NTSYS: Numerical taxonomy and multivariate analysis system
PCR: Polymerase chain reaction
QoIs: Quinone outside inhibitors
UPGMA: Unweighted pair group method with arithmetic mean
